# Holistic Processing for Other-Race Faces in Chinese Participants Occurs for Upright but Not Inverted Faces

**DOI:** 10.3389/fpsyg.2013.00029

**Published:** 2013-01-31

**Authors:** Kate Crookes, Simone Favelle, William G. Hayward

**Affiliations:** ^1^Department of Psychology, University of Hong KongHong Kong, China; ^2^ARC Centre of Excellence in Cognition and its Disorders and School of Psychology, The University of Western AustraliaPerth, WA, Australia; ^3^School of Psychology, University of WollongongWollongong, NSW, Australia

**Keywords:** holistic face processing, other-race effect, part-whole effect, inversion effect, face recognition

## Abstract

Recent evidence suggests stronger holistic processing for own-race faces may underlie the own-race advantage in face memory. In previous studies Caucasian participants have demonstrated larger holistic processing effects for Caucasian over Asian faces. However, Asian participants have consistently shown similar sized effects for both Asian and Caucasian faces. We investigated two proposed explanations for the holistic processing of other-race faces by Asian participants: (1) greater other-race exposure, (2) a general global processing bias. Holistic processing was tested using the part-whole task. Participants were living in predominantly own-race environments and other-race contact was evaluated. Despite reporting significantly greater contact with own-race than other-race people, Chinese participants displayed strong holistic processing for both Asian and Caucasian upright faces. In addition, Chinese participants showed no evidence of holistic processing for inverted faces arguing against a general global processing bias explanation. Caucasian participants, in line with previous studies, displayed stronger holistic processing for Caucasian than Asian upright faces. For inverted faces there were no race-of-face differences. These results are used to suggest that Asians may make more general use of face-specific mechanisms than Caucasians.

## Introduction

Faces from races with which we are familiar are easier to recognize than faces from races with which we have little experience. This own-race advantage (ORA) is a well-established phenomenon occurring across different countries and racial groups (see Meissner and Brigham, 2001, for review). However, the mechanisms underlying the ORA are less well understood.

Recent studies have suggested that weaker holistic processing for other-race than own-race faces might be a critical factor contributing to the ORA (Rhodes et al., 1989; Tanaka et al., 2004; Michel et al., 2006a,b). Holistic processing is a special perceptual mechanism used to encode upright faces, involving integration of information from across the whole-face (Maurer et al., 2002). Experimentally, holistic processing is measured using standard tasks including the part-whole task (Tanaka and Farah, 1993) and the composite task (Young et al., 1987). In the part-whole task participants learn a face, then at test are shown a pair of stimuli (a target and a foil) and are asked to recognize a feature from the studied face (e.g., the eyes). At test the features are presented either in isolation (part condition) or within the original face (whole condition). Foils in both the whole and the part condition differ from the studied face only by the target feature. Holistic processing is indicated by a memory advantage for the whole-face over the isolated part condition. Likewise, the composite task requires participants to identify one half of a face (e.g., the top-half) while ignoring the other half (e.g., the bottom-half). When the face halves are aligned to form a percept of a whole-face, interference from the bottom-half reduces identification accuracy for the top-half compared to when the halves are misaligned (i.e., do not form a single whole). In the general population these effects are observed for upright faces only and are absent or greatly reduced for other objects and inverted faces (for reviews see Rossion, 2008; McKone and Robbins, 2011; but see Richler et al., 2011b). Thus it is argued these tasks tap a style of processing that is specialized for upright faces.

While there is clear evidence for a holistic mode of processing for faces, are all faces processed holistically to the same degree? Three studies using the part-whole task with Caucasian participants have found a significant whole-face advantage for Caucasian but not Asian faces (Tanaka et al., 2004; Michel et al., 2006a; Mondloch et al., 2010), suggesting that holistic processing is used for own-race but not other-race faces. However, Asian participants have consistently shown a different pattern, that is, a whole-face advantage for both Asian and Caucasian faces with no significant interactions involving race-of-face (Tanaka et al., 2004; Michel et al., 2006a; Mondloch et al., 2010). Thus, these studies suggest that the race-of-participant may be a factor in determining whether the strength of holistic processing varies across different race faces.

Results from the composite task are mixed. The findings of Michel et al. (2006b) follow those for the part-whole effect; Caucasian participants showed a significant alignment disadvantage for Caucasian but not Asian faces, whereas Asian participants showed a significant alignment disadvantage for both Asian and Caucasian faces (although the effect was numerically larger for Asian faces). In contrast Mondloch et al. (2010) and Hayward et al. (in preparation) both found similar sized composite effects for Asian and Caucasian faces in both Asian and Caucasian participants suggesting no race-of-face effect on holistic processing.

One proposed explanation for the wider use of holistic processing is that Asian participants may have gained enough experience with Caucasians to process Caucasian faces holistically. This was certainly a possibility in two of the previous studies (Tanaka et al., 2004; Michel et al., 2006a) where the Asian participants were recruited and living in predominantly Caucasian countries. An alternative explanation for this lack of difference in holistic processing of own- and other-race faces is that Asians process all stimuli more globally than Caucasians (Michel et al., 2006a,b). Growing evidence from non-face domains suggests that Asians are more sensitive than Caucasians to the global context of a stimulus in perceptual tasks (e.g., center-surround size illusion, Doherty et al., 2008; rod-frame illusion, Ji et al., 2000; frame-line task, Kitayama et al., 2003). More directly, McKone et al. (2010) recently found that Asian participants showed a strong global bias relative to Caucasians on the Navon (1977) task suggesting a global bias in attention to objects. Thus it has been argued (Michel et al., 2006a,b) that the holistic processing observed for other-race faces in Asian participants might not result from face-specific mechanisms but rather from a domain-general global processing bias.

The aim of the present study was to investigate possible sources of the strong holistic processing of other-race faces by Asian participants. To perform this investigation we used the part-whole task with the same stimuli and general procedure as Tanaka et al. (2004). However, unlike that study, we used an Asian group living in a predominantly Asian country. We also evaluated participants’ experience with other-race individuals. We also added an inverted face condition. Inverted faces have traditionally been viewed as the ideal control for upright faces as they are identically matched on low-level image properties. No previous studies that we are aware of have tested Asian participants on the part-whole task with inverted stimuli. A finding of *no* significant part-whole effect for inverted faces in Asian participants would suggest that the task is tapping upright face-specific mechanisms rather than reflecting the general tendency to process all things more globally than Caucasians.

The addition of the inverted condition also allows us to control for other non-face-specific sources of the whole-face advantage. The part-whole task has received some criticism for not being a pure measure of holistic processing. It has been argued that some of the effect may be coming from generic context effects (i.e., better memory in the whole condition where the target feature appears in its original studied context than in the part condition where it is presented with no context, Gauthier and Tarr, 2002) or transfer-appropriate processing effects (i.e., the effect may be driven by a mismatch between study and test in the part condition; a reverse part-whole effect, a part advantage, has been observed when parts rather than whole-faces are presented in the study phase, Leder and Carbon, 2005). Such effects, if present, should also occur for inverted faces.

## Materials and Methods

### Participants

There were 38 Asian (24 female; mean age 23.1 years) and 38 Caucasian (30 female; mean age 21.7 years) participants. Asian participants were students or staff at the University of Hong Kong. All reported Chinese ancestry and China (Hong Kong/mainland) as their country of birth. None reported spending more than 6 months in a predominantly Caucasian country (e.g., UK). Caucasian participants were students at the University of Wollongong, Australia. The majority were born in Australia (1 England, 1 USA) and reported British or European ancestry. None reported spending more than 6 months in a predominantly Asian country. Participants received HK$30 (approximately US$4; Asian participants) or course credit (Caucasian participants) for the half hour experiment.

### Design

For Asian participants test condition (part, whole), race-of-face (Asian, Caucasian), and orientation (upright, inverted) were varied within-subjects. Orientation was blocked and the order of upright and inverted blocks was counterbalanced across participants. For Caucasian participants, test condition (part, whole) and race-of-face (Asian, Caucasian) were varied within-subjects. A programming error meant data were only available from the second orientation block for each participant therefore orientation (upright, inverted) was a between subjects factor. Where comparisons on the part-whole task were made between the Asian and Caucasian participants, only results from the second block of trials for each group were included, with orientation as a between subjects factor^1^.

### Materials

#### Face stimuli

**Figure 1:**
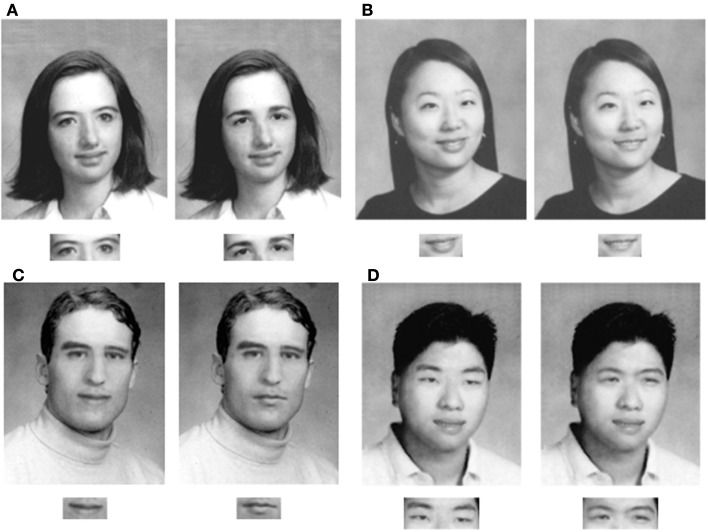
**Example whole-face and isolated part test pairs (A) Caucasian female, (B) Asian female, (C) Caucasian male, and (D) Asian male**. Whole foil faces differ from the target by only one feature. In the part condition only the critical feature was shown. A whole-face was always shown in the study phase of each trial.

The stimuli (Figure 1) were the same yearbook photographs of Caucasian and Asian males and females used by Tanaka et al. (2004).

There were 24 target faces: six Asian female, six Asian male, six Caucasian female, six Caucasian male. Within each of these sex and race categories a single face outline template was used. Each target face was created by pasting eyes, nose, and mouth features (taken from three different individuals) into the template.

Foils for the “whole” condition were created by swapping one feature (i.e., eyes, nose, or mouth) in the target face with that of another target face of the same race and sex. Target and foil stimuli for the “part” condition showed only the critical feature. Inverted stimuli were created by rotating each face by 180°.

At the viewing distance of approximately 60 cm whole-faces were an average of 5.1° vertical (hairline to chin) by 3.8° horizontal (cheek to cheek). For part trials the average sizes were: eyes 1.3° by 3.3°; noses 1.7° by 1.2°, and mouths 1.0° by 1.9°.

#### Racial background and contact questionnaire

Participants reported their racial background, place of birth, and time spent living abroad (including location). Level of interaction with both races was assessed using a questionnaire adapted from Hancock and Rhodes (2008)^2^. Participants rated their agreement with 14 statements on a 6-point scale (1 = very strongly disagree; 6 = very strongly agree). Half the statements were about contact with Asian people and the remaining half were the same statements but about Caucasians [e.g., “I socialize a lot with Asian (Caucasian) people”]. A higher score indicates a greater degree of contact.

### Procedure

The part-whole task was presented on a 17″ CRT screen (resolution 1024 × 768) using Psyscope X (Cohen et al., 1993; http://psy.ck.sissa.it/). Participants were tested individually.

The trial procedure was the same as that used by Tanaka et al. (2004) for their Asian participants. Each trial began with a fixation cross appearing at the center of the screen for 500 ms. The study face was then centrally presented for 500 ms followed by a scrambled face mask for 500 ms. The test pair, comprising the target face/feature and a foil face/feature, was then presented simultaneously approximately 11.4° apart until response. Participants pressed one key if the target was the left stimulus and another key for the right. A blank screen (1000 ms) followed each response.

Each orientation block comprised 144 trials. Each identity appeared as the target in six trials (three whole and three part), with each feature being the critical feature once in a whole trial and once in a part trial. Trial presentation order was randomized for each participant. The same trials were repeated, in different random order, in the upright, and inverted conditions. There was a 2 min break between orientation blocks.

The racial background and contact questionnaire followed the part-whole task.

## Results

### Contact

Self-reported contact with each race was calculated as the mean response to the seven statements regarding the particular race. An analysis of variance (ANOVA) with race-of-participant (Asian, Caucasian) as a between groups factor and race-of-contact (Asian, Caucasian) as a within-groups factor revealed a significant race-of-participant by race-of-contact interaction, *F*(1,74) = 292.41, MSE = 0.95, *p* < 0.001. Asian participants reported significantly greater contact with Asian (*M* = 5.5, SEM = 0.1) than Caucasian people (*M* = 2.3, SEM = 0.1), *t*(37) = 16.52, *p* < 0.001, correspondingly Caucasian participants reported significantly greater contact with Caucasian (*M* = 5.3, SEM = 0.1) than Asian people (*M* = 3.1, SEM = 0.2), *t*(37) = 8.93, *p* < 0.001.The ANOVA also revealed a significant main effects of race-of-contact, *F*(1,74) = 8.22, MSE = 0.95, *p* < 0.01, and race-of-participant, *F*(1,74) = 7.98, MSE = 0.42, *p* < 0.01, due to Caucasian participants reporting significantly greater contact with other-race people than Asian participants, *t*(74) = 3.20, *p* < 0.01.Reported contact with own-race people was not significantly different between the Asian and Caucasian groups, *t*(74) = 1.2, *p* > 0.2.

### Part-whole task: Accuracy by feature

Previous studies using the part-whole task have found performance on trials where the nose is the critical feature to be particularly poor compared to eyes and mouth trials (Tanaka and Farah, 1993; Pellicano and Rhodes, 2003). Initial screening of our data revealed that accuracy for nose trials was very poor. In Asian participants, collapsing across race-of-face, part-whole condition, and orientation mean accuracies were 74% for eyes, 68% for mouths, and 57% for noses. Similarly in Caucasian participants, collapsing across race-of-face and part-whole condition mean accuracies upright were 78% for eyes, 74% for mouths, and 61% for noses and inverted were 69% for eyes, 73% for mouths, and 55% for noses. We were concerned that such poor overall performance for noses may have led to a restriction of range problem for that feature, that is, performance so poor in the whole condition there is little room for the part condition to be significantly worse (for a discussion of this issue see McKone et al., 2012). If this were the case the overall size of the part-whole effect may be diluted by a lack of effect for noses.

For Asian participants, a repeated measures ANOVA with Feature (eyes, nose, mouth), Race-of-face (Asian, Caucasian), Part-whole Condition (part, whole), and Orientation (upright, inverted) revealed a significant main effect of Feature, *F*(2,74) = 86.29, MSE = 259.66, *p* < 0.001, and a significant Feature X Part-Whole Condition interaction, *F*(2,74) = 3.37, MSE = 222.33, *p* = 0.04, suggesting the size of the part-whole effect varied by feature. *A priori*, *t*-tests revealed that, for noses, there was no significant difference between whole and part conditions for Asian or Caucasian faces upright or inverted (all *p*s > 0.2). Similarly for Caucasian participants a mixed ANOVA with Orientation as a between subjects variable also found a significant main effect of Feature, *F*(2,72) = 31.37, MSE = 290.33, *p* < 0.001, and a significant Feature X Part-Whole Condition interaction, *F*(2,72) = 5.63, MSE = 191.00, *p* = 0.005, and *t*-tests showed no significant difference on nose trials between whole and part conditions for Asian or Caucasian faces upright or inverted (all *t*s < 1). Overall there was no indication of a part-whole effect for nose trials. We therefore decided to exclude nose trials from further analysis. The same general patterns of part-whole results were observed when nose trials were included however fewer effects were significant. It should be noted that previous studies have not always included all features in analyses, Michel et al. (2006a) looked at the part-whole effect for eye trials only (nose and mouth trials were included in the experiment as “catch” trials).

### Part-whole effect: Asian participants

#### Accuracy

**Figure 2:**
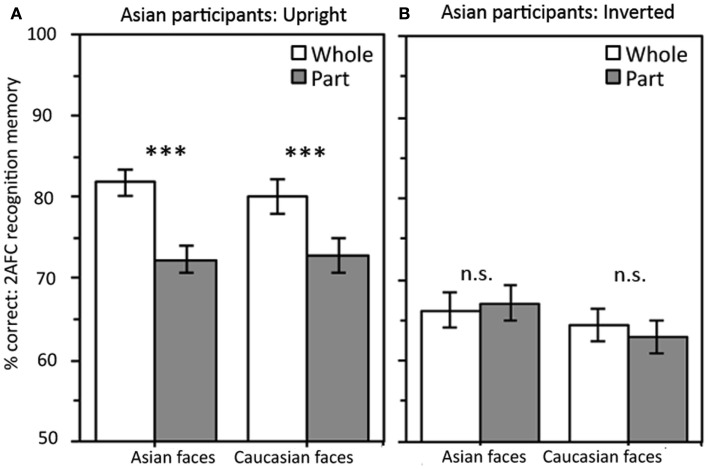
**Asian participants show: (A) significant whole-face advantage in the upright condition for both Asian and Caucasian faces; and (B) no evidence of holistic processing for inverted faces of either race**. Error bars are as appropriate for making the within-subjects comparison between whole and part conditions (i.e., ±1 SEM of the whole – part difference scores). ***p ≤ 0.001.

Our primary interest was whether Asian participants processed both own- and other-race faces holistically and, if so, whether this holistic processing was also present for inverted faces. The accuracy results presented in Figure 2 show a strong whole over part advantage in the upright condition for both Asian and Caucasian faces, whereas inverted there was no sign of a part-whole effect for either race-of-face. This was supported by a repeated measures ANOVA with the variables Part-Whole Condition (whole, part), Race-of-Face (Asian, Caucasian), and Orientation (upright, inverted) which revealed significant main effects of Part-Whole Condition, *F*(1,37) = 21.67, MSE = 66.61, *p* < 0.001, and Orientation, *F*(1,37) = 63.81, MSE = 158.64, *p* < 0.001, and importantly a significant Part-Whole Condition X Orientation interaction, *F*(1,37) = 14.57, MSE = 84.71, *p* < 0.001.The main effect of Race-of-Face and all interactions involving Race-of-Face were not significant, all *F*s < 2.6, all *p*s > 0.12. Further analyses were conducted on the upright and inverted conditions separately.

For *upright* faces (Figure 2A) a Part-Whole Condition X Race-of-Face repeated measures ANOVA produced a significant main effect of Part-Whole Condition, *F*(1,37) = 33.47, MSE = 79.88, *p* < 0.001, and follow up *t*-tests showed the whole over part advantage was significant for both Asian faces, *t*(37) = 5.73, *p* < 0.001, and Caucasian faces, *t*(37) = 3.47, *p* = 0.001.There was no significant main effect of Race-of-Face, *F* < 1, MSE = 71.85 or interaction between Part-Whole Condition and Race-of-Face, *F* < 1, MSE = 56.72, confirming that the size of the part-whole effect (whole minus part) was not larger for own-race (9.4%) than other-race (7.3%) faces.

For inverted faces (Figure 2B), a Part-Whole Condition by Race-of-Face ANOVA revealed no significant main effects or interaction, all *F*s < 3.6, all *p*s > 0.06.Asian participants showed no whole-face advantage for either Asian inverted faces, *t* < 1, or Caucasian inverted faces, *t* < 1. There was no evidence that Asian participants processed inverted faces holistically^3^.

#### Reaction time

Participants were not instructed to respond as quickly as possible however to ensure there were no speed-accuracy trade offs median reaction times (RTs) for correct eyes and mouth trials for each condition were calculated for each participant. A repeated measures ANOVA with variables Part-Whole Condition (whole, part), Race-of-Face (Asian, Caucasian), and Orientation (upright, inverted) produced a main effect of Race-of-Face, *F*(1,37) = 5.59, MSE = 63446.97, *p* = 0.023, with faster responses for Asian faces (*M* = 1466 ms) than Caucasian faces (*M* = 1534 ms). There was also a significant main effect of Orientation, *F*(1,37) = 14.46, MSE = 293673.59, *p* = 0.001, with faster responses upright (*M* = 1382 ms) than inverted (*M* = 1618 ms). While the main effect of Part-Whole Condition was only marginally significant, *F*(1,37) = 3.75, MSE = 161151.68, *p* = 0.060, there was a significant Orientation by Part-Whole Condition interaction, *F*(1,37) = 30.71, MSE = 112415.92, *p* < 0.001. All remaining interactions were non-significant, all *F*s < 1. For upright trials a Part-Whole Condition X Race-of-Face repeated measures ANOVA again revealed a significant main effect of Race-of-Face, *F*(1,37) = 8.96, MSE = 17678.47, *p* = 0.005, indicating faster response for Asian (*M* = 1350) than Caucasian faces (*M* = 1414). There was also a significant main effect of Part-Whole Condition, *F*(1,37) = 5.00, MSE = 116707.89, *p* = 0.031, with longer RTs to Part (*M* = 1444) than Whole trials (*M* = 1320). The interaction was not significant, *F*(1,37) = 1.20, MSE = 26921.78, *p* > 0.2.Therefore, there was no indication of a speed-accuracy trade off in the critical upright face condition. For inverted faces the same analysis produced a main effect of Part-Whole Condition, *F*(1,37) = 22.14, MSE = 156859.72, *p* < 0.001, however in this case there was a reverse part-whole effect, that is, RTs were longer for Whole (*M* = 1769) than Part (*M* = 1467) trials. There was no significant main effect of Race-of-Face, *F*(1,37) = 1.79, MSE = 110056.78, *p* > 0.1, or interaction, *F* < 1, MSE = 80276.02.

### Part-whole effect: Caucasian participants

#### Accuracy

**Figure 3:**
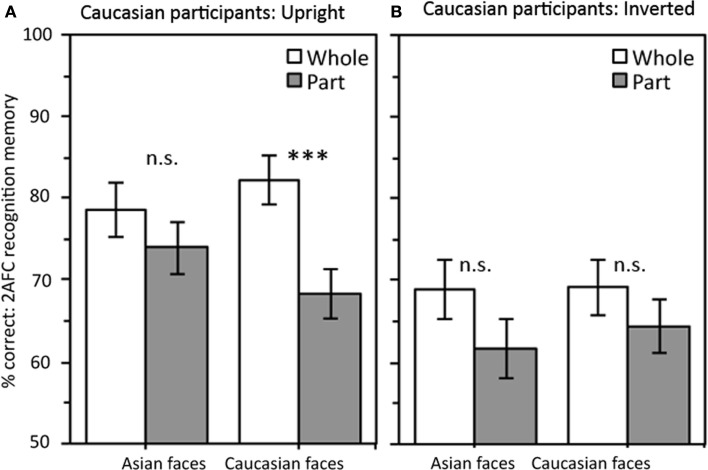
**Results for Caucasian participants**. **(A)** For upright faces a significant whole-face advantage was observed for Caucasian faces only. **(B)**. There was no significant part-whole effect for either race-of-face when faces were inverted. Note orientation was varied between subjects for the Caucasian participants. Error bars are as appropriate for making the within-subjects comparison between whole and part conditions (i.e., ±1 SEM of the whole – part difference scores). ***p < 0.001.

The accuracy results shown in Figure 3 suggest Caucasian participants, unlike the Asian participants, show a stronger whole-face advantage for own-race than other-race upright faces. An ANOVA with Part-Whole Condition and Race-of-Face as repeated measures factors and Orientation as a between groups factor produced significant main effects of Part-Whole Condition, *F*(1,36) = 19.16, MSE = 115.17, *p* < 0.001, and Orientation, *F*(1,36) = 12.34, MSE = 290.38, *p* = 0.001, and a three-way interaction that approached significance, *F*(1,36) = 3.62, MSE = 95.50, *p* = 0.065. The main effect of Race-of-Face and remaining interactions were not significant, all *p*s > 0.2.

As we did for the Asian participants we analyzed the two orientations separately. For upright faces (Figure 3A) there was a significant main effect of Part-Whole condition, *F*(1,18) = 15.89, MSE = 103.86, *p* = 0.001. This was qualified by a significant interaction between Race-of-Face and Part-Whole Condition, *F*(1,18) = 5.19, MSE = 81.42, *p* = 0.035, confirming the part-whole effect was significantly larger for Caucasian faces (14.0%) than Asian faces (4.6%). Follow up *t*-tests found a significant whole-face advantage for Caucasian faces, *t*(18) = 4.69, *p* < 0.001, but not Asian faces, *t*(18) = 1.42, *p* > 0.1.

In the inverted orientation (Figure 3B) a Part-Whole Condition by Race-of-Face ANOVA revealed no significant main effect of race-of-face, *F* < 1, MSE = 59.72, or interaction, *F* < 1, MSE = 109.57. There was a main effect of part-whole condition, *F*(1,18) = 5.27, MSE = 126.48, *p* = 0.034 but the part-whole effect was not significant for either race-of-face considered alone: Caucasian faces [4.6%, *t*(18) = 1.38, *p* > 0.1], Asian faces [7.2%, *t*(18) = 1.96, *p* > 0.06]. *Post hoc* independent samples *t*-tests showed that for Caucasian faces the part-whole effect was significantly stronger for upright than inverted faces, *t*(36) = 2.10, *p* = 0.043, for Asian faces there was a non-significant trend in the reverse direction, *t* < 1.

#### Reaction time

An ANOVA on median RTs for correct eye and mouth trials with Part-Whole Condition and Race-of-Face as repeated measures factors and Orientation as a between groups factor revealed only a significant main effect of Race-of-Face, *F*(1,36) = 7.03, MSE = 28896.29, *p* = 0.012, with faster RTs for Asian (*M* = 1457 ms) than Caucasian faces (*M* = 1530). All other main effects and interactions were not significant, all *F*s < 1.9, all *p*s > 0.1.In the upright group there was no indication of a speed-accuracy trade off, since the trends in RT matched those seen for accuracy. Inverted, the pattern was similar to that shown by the Asian participant group – a trend for longer RTs for whole compared to part trials.

### Part-whole effect: Comparing Asian and Caucasian participants

**Figure 4:**
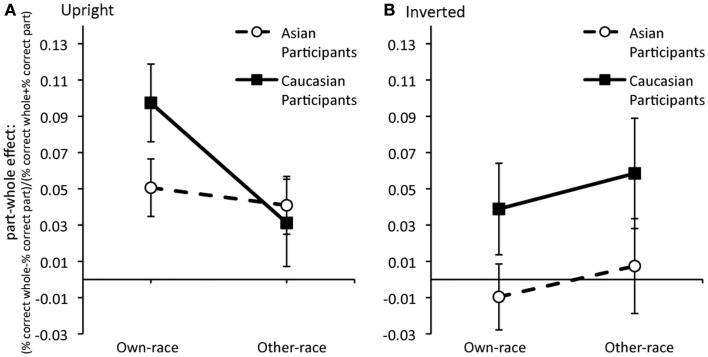
**Standardized part-whole effect (% correct whole − % correct part)/(% correct whole + % correct part) for (A) upright and (B) inverted own-race and other-race faces**. Error bars are ±1 SEM.

Despite the suggestion of different patterns between the Caucasian and Asian participants analyses of the second block of trials did not produce statistically significant differences. To control for any differences in baseline accuracy standardized part-whole scores [(% correct whole − % correct part)/(% correct whole + % correct part)] were calculated for own- and other-race faces (Figure 4). A mixed ANOVA with Face Category (own, other) as a within-subjects variable and Orientation (upright, inverted) and Race-of-Participant (Asian, Caucasian) produced only a main effect of Race-of-Participants, *F*(1,72) = 4.53, MSE = 0.010, *p* = 0.037, reflecting larger part-whole effects for Caucasian than Asian participants. The main effect of Orientation was marginally significant *F*(1,72) = 3.79, MSE = 0.010, *p* = 0.056, suggesting stronger holistic processing for upright than inverted faces. The critical three-way interaction was not significant, *F* < 1, MSE = 0.010, nor were the main effect of Face Category and all other interactions, *p*s > 0.08.

## Discussion

Our main finding was that Asian participants showed strong holistic processing for upright own-race and other-race faces, and the part-whole effect was not weaker for other-race faces. This replicates a previous finding using the same stimuli in Asian participants living in Canada who reported no difference in contact with own- and other-race people (Tanaka et al., 2004). In the present study participants were living in a predominantly own-race environment and reported greater contact with own-race people. This finding also agrees with other previous demonstrations of strong holistic processing for other-race faces in Asian participants using different stimuli (Michel et al., 2006a; Mondloch et al., 2010) and methods (i.e., composite effect Michel et al., 2006b; Mondloch et al., 2010). Furthermore, there are no studies to the contrary, in that no previous studies have reported significantly stronger holistic processing for own-race over other-race faces in Asian participants. Importantly, we also found no indication of holistic processing for either race of inverted faces in Asian participants. This finding suggests that holistic processing of upright other-race faces is not the result of a tendency of Asian participants to process all stimuli globally. We are not aware of any previous studies that have tested holistic processing for inverted faces in Asian participants.

A different pattern was observed for Caucasian participants who, as in previous studies, showed stronger holistic processing for upright own-race faces than other-race faces. This result supports previous findings using these stimuli (Tanaka et al., 2004), different stimuli (Michel et al., 2006a; Mondloch et al., 2010), and different methods (Michel et al., 2006b). Although there was a significant part-whole effect for inverted faces with Caucasian participants, the effect was relatively weak, was not statistically significant for either race-of-face by itself, and was not statistically different from the lack of part-whole effect observed for inverted faces with Asian participants. Furthermore, previous studies have occasionally found significant part-whole effects for inverted faces (Palermo and Rhodes, 2002; Boutet and Faubert, 2006) possibly stemming from non-face-specific context effects (i.e., more change to the image between study and test in the part condition than the whole condition leading to poorer performance in the part condition). Overall, therefore, the most theoretically important finding for Caucasian participants was that the part-whole effects for inverted faces were predictably much smaller than that seen for own-race upright faces.

These race differences need to be interpreted with caution given the lack of significant effects when directly comparing the two participant groups, however, this pattern of results supports previous findings of a difference between Asian and Caucasian participants in the holistic processing of other-race faces (Tanaka and Farah, 1993; Michel et al., 2006a,b; Mondloch et al., 2010). Importantly, the findings of the present study argue against two previous explanations of the holistic processing of other-race faces observed for Asian participants.

First, in two previous studies (Tanaka et al., 2004; Michel et al., 2006a) Asian participants had substantially more other-race contact than the Caucasian participants suggesting that experience with other-race faces could explain the presence of holistic processing. Greater contact with other-race individuals has been associated with a reduction in the ORA in memory (for review see Meissner and Brigham, 2001, although not always, e.g., deHeering et al., 2010) and the ORA in size of the inversion effect (Hancock and Rhodes, 2008). Here we attempted to minimize differences between the groups in other-race contact by testing participants living in predominantly own-race environments but still found holistic processing of other-race faces by Asian participants. Moreover, both Asian and Caucasian participants reported greater in-person contact with own-race than other-race people yet only the Caucasian group showed greater holistic processing for own-race faces. These results suggest that amount of contact is not the exclusive source of holistic processing of other-race faces. However we did not assess the quality of in-person contact (Bukach et al., 2012) or exposure to other-race faces in the media. Future studies using another race-of-faces other than Caucasian as the other-race face stimuli (e.g., African faces) may strengthen these conclusions.

If not amount of contact, what other factors might influence the presence of holistic processing for other-race faces? The age at which experience with other-race faces is gained may be an important factor. Recent studies have shown that the ORA in recognition is present in children as young as 6 months (Kelly et al., 2007, 2009; Heron-Delaney et al., 2011). However if infants are exposed to other-race faces, then discrimination ability for other-race faces is maintained at 9 months (Heron-Delaney et al., 2011). It could be that holistic processing is observed for other-race faces if some amount of contact is gained within a sensitive period early in development. Another factor possibly influencing holistic processing of other-race faces is social categorization. Michel et al. (2007, 2010) have shown that the strength of holistic processing for ambiguous race faces (i.e., a morph between a Caucasian and an Asian face) can be influenced by changing the perceived race, that is, the composite effect is larger when an ambiguous face is perceived as own-race than when the same face is perceived as other-race. It could be that holistic processing is observed for other-race faces if they are categorized as “own-group” in some way.

Second, this study provides the first demonstration that holistic processing of other-race faces by Asian participants may not result from a domain-general global processing bias. Asian participants showed strong holistic processing for both Asian and Caucasian *upright* faces, but there was no evidence of holistic processing for inverted faces. Recent studies have suggested that holistic processing might sometimes occur for inverted faces and that therefore inverted faces may not be a pure control stimulus (Richler et al., 2011a). However contrary to this we found no indication of holistic processing as measured by the part-whole effect for inverted faces in our Asian participant group. Thus, rather than Asian participants exhibiting a general tendency to process all stimuli globally, holistic processing was limited to upright faces suggesting face-specific mechanisms as the origin. Further, our analysis comparing the two groups of participants found Caucasian participants overall displayed significantly stronger holistic processing than Asian participants.

Together with previous findings our results suggest wider use of face-specific mechanisms by Asian participants. There appears to be a difference between Asians and Caucasians in the degree of variability tolerated by face processing mechanisms, that is, for Caucasians holistic processing as measured by the part-whole effect is more strongly activated for own-race faces, whereas for Asians holistic processing can be used for face types beyond those commonly experienced. Alternatively, rather than reflecting a face-specific phenomena, it could be that Asians show greater global processing for objects in their canonical orientation. Future studies with Asian participants using non-face stimuli that are not processed holistically by Caucasian participants (e.g., Labrador dogs, Robbins and McKone, 2007) would test this possibility.

### Origins of the ORA in recognition memory

The consistent finding that Asian participants show holistic processing for other-race faces brings in to question the claim that the ORA for recognition results from weak holistic processing of other-race faces. While the present study did not test the ORA in memory, previous studies have found a memory advantage for own-race faces in Asians in Hong Kong and Caucasians in Australia (Rhodes et al., 2006; Hayward et al., 2008). Our holistic processing results are also consistent with those of previous studies that have found an ORA in Asian participants despite not finding a race-of-face effect for holistic processing (Michel et al., 2006a,b). Indeed, these latter studies also demonstrated a lack of a correlation between the ORA and the holistic processing advantage for own-race faces in Caucasian participants, suggesting that more generally the strength of holistic processing may be related only indirectly to differences in recognition memory ability for own- and other-race faces.

Here we have considered only one aspect of the “special” processing of faces. While holistic processing deficits for other-race faces might not be the source of the ORA, tasks tapping different aspects of face processing do show complementary effects in Asian and Caucasian participants. Rhodes et al. (1989)and Hancock and Rhodes (2008) demonstrated larger inversion effects for own-race than other-race faces in both groups. Inversion not only disrupts holistic processing but also configural (spacing between the features) and component (feature shape) processing (see McKone and Yovel, 2009, for review). Tasks directly tapping configural and component processing reliably produced larger effects for own-race faces in both Asian and Caucasian participants (Rhodes et al., 2006, 2009; Hayward et al., 2008; Mondloch et al., 2010), suggesting that poor sensitivity to aspects of face-specific processing other than holistic processing might underlie the ORA.

### Implications for face recognition

This discussion has highlighted a gap in the understanding of the relationship between holistic processing and face recognition ability. Rather than being something that everybody is good at, recent evidence suggests that within the general population there exists a broad range of face recognition abilities (e.g., Bowles et al., 2009). The recent finding that congenital prosopagnosics who, by definition, display poor face recognition also show weaker holistic processing, suggests there is a link between holistic processing and recognition (Palermo et al., 2011). Early studies at an individual differences level have produced mixed results, while some researchers have found a relationship between holistic processing and recognition (Richler et al., 2011a; Wang et al., 2012), others have not (Konar et al., 2010). Studies investigating race-of-face effects from an individual differences perspective may help clarify the relationship between measures of holistic/configural processing and recognition ability.

## Conflict of Interest Statement

The authors declare that the research was conducted in the absence of any commercial or financial relationships that could be construed as a potential conflict of interest.

## Footnotes

^1^The Asian participants’ results for the second condition alone followed the same pattern as those in the full within-subjects design.

^2^We used the more general term “Asian” rather than “Chinese” on the questionnaire as the ethnicity of the face stimuli used was not specified by Tanaka et al. (2004) and it is unlikely that our Caucasian participants were aware of the exact ethnic background of every Asian person they have encountered.

^3^To explore the possibility that a floor effect masked a part-whole effect in the inverted condition we ran 10 additional Asian participants on the inverted task with extended study duration (1000 ms) to boost accuracy. Overall mean accuracy for eyes and mouth trials was 73%. The whole-face advantage was again small and non-significant for both Asian (*M* = 0.8, *t* < 1) and Caucasian faces (*M* = 2.1, *t* < 1).

## References

[B1] Boutet I. Faubert J. (2006). Recognition of faces and complex objects in younger and older adults. Mem. Cogn. 34, 854–864 10.3758/BF03193432 17063916

[B2] Bowles D. C. McKone E. Dawel A. Duchaine B. C. Palermo R. Schmalzl L. (2009). Diagnosing prosopagnosia: effects of ageing, sex, and participant-stimulus ethnic match on the Cambridge Face Memory Test and Cambridge Face Perception Test. Cogn. Neuropsychol. 25, 423–455 10.1080/02643290903343149 19921582

[B3] Bukach C. Cottle J. Ubiwa J. Miller J. (2012). Individuation experience predicts other-race effects in holistic processing for both Caucasian and Black participants. Cognition 123, 319–343 10.1016/j.cognition.2012.02.007 22397819

[B4] Cohen J. MacWhinney B. Flatt M. Provost P. (1993). PsyScope: an interactive graphic system for designing and controlling experiments in the psychology laboratory using Macintosh computers. Behav. Res. Methods Instrum. Comput. 25, 257–271 10.3758/BF03204507

[B5] deHeering A. de Liedekerke C. Deboni M. Rossion B. (2010). The role of experience during childhood in shaping the other-race effect. Dev. Sci. 13, 181–187 10.1111/j.1467-7687.2009.00876.x 20121874

[B6] Doherty M. J. Tsuji H. Phillips W. A. (2008). The context sensitivity of visual size perception varies across cultures. Perception 37, 1426–1433 10.1068/p5946 18986068

[B7] Gauthier I. Tarr M. J. (2002). Unraveling mechanisms for expert object recognition: bridging brain activity and behavior. J. Exp. Psychol. Hum. Percept. Perform. 28, 431–446 10.1037/0096-1523.28.2.431 11999864

[B8] Hancock K. J. Rhodes G. (2008). Contact, configural coding and the other-race effect in face recognition. Br. J. Psychol. 99, 45–56 10.1348/000712607X199981 17535471

[B9] Hayward W. G. Rhodes G. Schwaninger A. (2008). An own-race advantage for components as well as configurations in face recognition. Cognition 106, 1017–1027 10.1016/j.cognition.2007.04.002 17524388

[B10] Heron-Delaney M. Anzures G. Herbert J. Quinn P. Slater A. Tanaka J. (2011). Perceptual training prevents the emergence of the other race effect during infancy. PLoS ONE 6:e19858 10.1371/journal.pone.0019858 21625638 PMC3097220

[B11] Ji L.-J. Peng K. Nisbett R. E. (2000). Culture, control, and perception of relationships in environment. J. Pers. Soc. Psychol. 78, 943–955 10.1037/0022-3514.78.5.943 10821200

[B12] Kelly D. J. Liu S. Lee K. Quinn P. C. Pascalis O. Slater A. M. (2009). Development of the other-race effect during infancy: evidence toward universality? J. Exp. Child. Psychol. 104, 105–114 10.1016/j.jecp.2009.01.006 19269649 PMC3740564

[B13] Kelly D. J. Quinn P. C. Slater A. M. Lee K. Ge L. Pascalis O. (2007). The other-race effect develops during infancy: evidence of perceptual narrowing. Psychol. Sci. 18, 1084–1089 10.1111/j.1467-9280.2007.02029.x 18031416 PMC2566514

[B14] Kitayama S. Duffy S. Kawamura T. Larsen J. T. (2003). Perceiving an object and its context in different cultures: a cultural look at new look. Psychol. Sci. 14, 201–206 10.1111/1467-9280.02432 12741741

[B15] Konar Y. Bennett P. J. Sekuler A. B. (2010). Holistic processing is not correlated with face-identification accuracy. Psychol. Sci. 21, 38–43 10.1177/0956797609356508 20424020

[B16] Leder H. Carbon C.-C. (2005). When context hinders! Learn-test compatibility in face recognition. Q. J. Exp. Psychol. 58, 235–250 10.1080/02724980343000936 15903116

[B17] Maurer D. Le Grand R. Mondloch C. J. (2002). The many faces of configural processing. Trends Cogn. Sci. (Regul. Ed.) 6, 255–260 10.1016/S1364-6613(02)01903-4 12039607

[B18] McKone E. Aimola Davies A. Fernando D. Aalders R. Leung H. Wickramariyaratne T. (2010). Asia has the global advantage: race and visual attention. Vision Res. 50, 1540–1549 10.1016/j.visres.2010.05.010 20488198

[B19] McKone E. Crookes K. Jeffery L. Dilks D. D. (2012). A critical review of the development of face recognition: experience is less important than previously believed. Cogn. Neuropsychol. 29, 174–212 10.1080/02643294.2012.660138 22360676

[B20] McKone E. Robbins R. (2011). “Are faces special?” in The Oxford Handbook of Face Perception, eds Calder A. J. Rhodes G. Johnson M. H. Haxby J. V. (Oxford: Oxford University Press), 149–176

[B21] McKone E. Yovel G. (2009). Why does picture-plane inversion sometimes dissociate perception of features and spacing in faces, and sometimes not? Toward a new theory of holistic processing. Psychon. Bull. Rev. 16, 778–797 10.3758/PBR.16.5.778 19815781

[B22] Meissner C. A. Brigham J. C. (2001). Thirty years of investigating the own-race bias in memory for faces. Psychol. Publ. Pol. Law 7, 3–35 10.1037/1076-8971.7.1.3

[B23] Michel C. Caldara R. Rossion B. (2006a). Same-race faces are perceived more holistically than other-race faces. Vis. Cogn. 14, 55–73 10.1080/13506280500158761

[B24] Michel C. Rossion B. Han J. Chung C.-S. Caldara R. (2006b). Holistic processing is finely tuned for face of one’s own race. Psychol. Sci. 17, 608–615 10.1111/j.1467-9280.2006.01752.x 16866747

[B25] Michel C. Corneille O. Rossion B. (2007). Race categorization modulates holistic face encoding. Cogn. Sci. 31, 911–924 10.1080/03640210701530805 21635322

[B26] Michel C. Corneille O. Rossion B. (2010). Holistic face encoding is modulated by perceived face race: evidence from perceptual adaptation. Vis. Cogn. 18, 434–455 10.1080/13506280902819697

[B27] Mondloch C. J. Elms N. Maurer D. Rhodes G. Hayward W. G. Tanaka J. W. (2010). Processes underlying the cross-race effect: an investigation of holistic, featural, and relational processing of own- vs. other-race faces. Perception 39, 1065–1085 10.1068/p6153 20942358

[B28] Navon D. (1977). Forest before trees: the precedence of global features in visual perception. Cogn. Psychol. 9, 353–383 10.1016/0010-0285(77)90012-3

[B29] Palermo R. Rhodes G. (2002). The influence of divided attention on holistic face perception. Cognition 82, 225–257 10.1016/S0010-0277(01)00160-3 11747863

[B30] Palermo R. Willis M. L. Rivolta D. McKone E. Wilson C. E. Calder A. J. (2011). Impaired holistic coding of facial expression and facial identity in congenital prosopagnosia. Neuropsychologia 49, 1226–1235 10.1016/j.neuropsychologia.2011.02.021 21333662 PMC3083514

[B31] Pellicano E. Rhodes G. (2003). Holistic processing of faces in preschool children and adults. Psychol. Sci. 14, 618–622 10.1046/j.0956-7976.2003.psci_1474.x 14629695

[B32] Rhodes G. Brake S. Taylor K. Tan S. (1989). Expertise and configural coding in face recognition. Br. J. Psychol. 80, 313–331 10.1111/j.2044-8295.1989.tb02323.x 2790391

[B33] Rhodes G. Ewing L. Hayward W. G. Maurer D. Mondloch C. J. Tanaka J. W. (2009). Contact and other-race effects in configural and component processing of faces. Br. J. Psychol. 100, 717–728 10.1348/000712608X396503 19228441

[B34] Rhodes G. Hayward W. G. Winkler C. (2006). Expert face coding: configural and component coding of own-race and other-race faces. Psychon. Bull. Rev. 13, 499–505 10.3758/BF03193876 17048737

[B35] Richler J. J. Cheung O. S. Gauthier I. (2011a). Holistic processing predicts face recognition. Psychol. Sci. 22, 464–471 10.1177/0956797611401753 21393576 PMC3077885

[B36] Richler J. J. Mack M. L. Palmeri T. J. Gauthier I. (2011b). Inverted faces are (eventually) processed holistically. Vision Res. 51, 333–342 10.1016/j.visres.2010.11.014 21130798

[B37] Robbins R. McKone E. (2007). No face-like processing for objects-of-expertise in three behavioural tasks. Cognition 103, 34–79 10.1016/j.cognition.2006.02.008 16616910

[B38] Rossion B. (2008). Picture-plane inversion leads to qualitative changes of face perception. Acta Psychol. (Amst.) 128, 274–289 10.1016/j.actpsy.2008.02.003 18396260

[B39] Tanaka J. W. Farah M. J. (1993). Parts and wholes in face recognition. Q. J. Exp. Psychol. 46A, 225–245 10.1080/14640749308401045 8316637

[B40] Tanaka J. W. Kiefer M. Bukach C. M. (2004). A holistic account of the own-race effect in face recognition: evidence from a cross-cultural study. Cognition 93, B1–B9 10.1016/j.cognition.2003.10.002 15110726

[B41] Wang R. Li J. Fang H. Tian M. Liu J. (2012). Individual differences in holistic processing predict face recognition ability. Psychol. Sci. 23, 169–177 10.1177/0956797611420575 22222218

[B42] Young A. W. Hellawell D. Hay D. C. (1987). Configurational information in face perception. Perception 16, 747–759 10.1068/p160747 3454432

